# Diet-induced microbial adaptation process of red deer (*Cervus elaphus*) under different introduced periods

**DOI:** 10.3389/fmicb.2022.1033050

**Published:** 2022-10-20

**Authors:** Jinhao Guo, Yongchao Jin, Xinmin Tian, Heng Bao, Yue Sun, Thomas Gray, Yaqi Song, Minghai Zhang

**Affiliations:** ^1^College of Wildlife and Protected Area, Northeast Forestry University, Harbin, China; ^2^World Wild Fund for Nature (China), Changchun, China; ^3^College of Life Science and Technology, Mudanjiang Normal University, Mudanjiang, China; ^4^School of Biological Sciences, Guizhou Education University, Guiyang, China; ^5^WWF Tigers Alive Initiative, Singapore, Singapore

**Keywords:** red deer, introduction, dietary, gut microbes, adaptation

## Abstract

Insufficient prey density is a major factor hindering the recovery of the Amur tiger (*Panthera tigris altaica*), and to effectively restore the Amur tiger, red deer (*Cervus elaphus*) was released into the Huangnihe National Nature Reserve of Northeast China as the main reinforcement. Differences in feeding and synergistic changes caused by the intestinal microbial communities could impact the adaptation of wildlife following reintroductions into field environments. We analyzed the foraging changes in shaping the intestinal microbial community of the red deer after being released to the Huangnihe National Nature Reserve and screened the key microbial flora of the red deer when processing complex food resources. The feeding and intestinal microbial communities of the red deer were analyzed by plant Deoxyribonucleic acid (DNA) barcoding sequencing and 16S rRNA high-throughput sequencing, respectively. The results showed that there were significant differences in food composition between wild and released groups [released in 2019 (R2): *n* = 5; released in 2021 (R0): *n* = 6]; the wild group fed mainly on *Acer* (31.8%) and *Abies* (25.6%), R2 fed mainly on *Betula* (44.6%), R0 had not formed a clear preferred feeding pattern but had certain abilities to process and adapt to natural foods. Firmicutes (77.47%) and Bacteroides (14.16%) constituted the main bacterial phylum of red deer, of which, the phylum Firmicutes was the key species of the introduced red deer for processing complex food resources (*p* < 0.05). The wild release process significantly changed the intestinal microbial structure of the red deer, making it integrate into the wild red deer. The period since release into the wild may be a key factor in reshaping the structure of the microbial community. This study suggested that the intestinal microbial structure of red deer was significantly different depending on how long since captive deer has been translocated. Individuals that have lived in similar environments for a long time will have similar gut microbes. This is the adaption process of the wildlife to natural environment after wild release, taking into account the gut microbes, and the feeding changes in shaping microbial communities can help introduced red deer match complex food resources and novel field environments.

## Introduction

Wildlife reintroductions and translocations are important conservation methodologies, and conservation translocation is the intentional movement and release of living organisms within or outside their indigenous range to improve the conservation status of the focal species (IUCN/SSC, 2013). Effective introduction can increase the number of endangered animals and avoid extinction in the wild. The red deer (*Cervus elaphus*) is a typical forest-inhabiting mammal in northeast China and has an important ecological status in the forest ecosystem (Wang, 1998). However, due to logging before the 1980s and frequent human activities, the distribution range of red deer had been gradually shrinking, and the population number had decreased by > 70% at the end of the last century (Xu et al., 2000). The decline in red deer abundance not only affects the health and stability of the ecosystem but also impacts the recovery of large carnivores, especially the Amur tiger (*Panthera tigris altaica*) (Sun, 2015; Gu et al., 2018). Reduced deer abundance is an important limitation for Amur tiger recovery (Soh et al., 2014; Qi et al., 2015; Niu et al., 2021). Therefore, from the perspective of protecting the red deer, and the perspective of increasing the prey density of the Amur tiger, the population reinforcement of red deer has practical biological conservation significance.

Conservation translocations from captivity to the wild force animals to confront new environment and process new food resources. The study on the adaptability of introduced sika deer (*Cervus nippon*) has found drastic changes in food composition in the early stage of releasing period, and the stabilized feeding habits gradually formed in the late stage of releasing period and were similar to the natural populations (Shi, 2016). Released sika deer gradually change from opportunistic random feeding to selective feeding, which is the process of feeding adaptation of sika deer to the natural environment. In translocation of captive-bred North-African houbara bustards (*Chlamydotis undulata undulata*), the released individuals had good adaptability to natural diets with similar food composition to wild groups (Bourass and Hingrat, 2015). However, the reintroductions and translocations for conservation purposes often have a lower success rate (Fischer and Lindenmayer, 2000; Mathews et al., 2005; Jule et al., 2008), and any feeding disorder may lead to the failure of released works. It is necessary to study the adaptability of released animals to food in the natural environment, and this is what the IUCN guidelines of reintroductions and translocations advocate (IUCN/SSC, 2013).

The gut microbiome of wildlife is not only closely related to the requirements of nutrition and health but also affected by food and habitats (Ley et al., 2008; Wei et al., 2019). Animals living in different habitats have different intestinal flora with different diets (Rothschild et al., 2017; Li et al., 2021; Xia et al., 2021). Animals have to adapt to changes in habitats and food after introduction, which can lead to synergistic changes in the gut microbiome. The feeding composition of wooly monkeys (*Lagothrix lagothricha*) changed significantly after they were released into the wild, and the microbial structure and function were reshaped to varying degrees within days after release (Quiroga-González et al., 2021). The effects of food and habitat on gut microbes can also be found in captivity. Compared to the captive individuals, the feeding and microbial communities of introduced Przewalski’s horses (*Equus ferus*) changed significantly (Li Y. et al., 2019). Numerous studies have been conducted on the gut microbiota of wild and captive animals, and it has been proven that captive environments contribute to differences in food and microbial communities (Guan et al., 2017; Li et al., 2017). It follows that food and the environment can drive changes in the gut microbiome. For the released red deer, the gut microbiome is likely to play an important role, especially in the adaption to complex survival conditions and food resources in the wild. Currently, the distribution and the differences related to the microbial community between the introduced and the wild red deer are not clear, and the factors driving the changes in gut microbes are still unknown; the ecological role played by intestinal microbiota in the wild release process needs to be further studied, and the complex mechanism between red deer-environment-intestinal microorganisms needs to be explored, which will provide a theoretical reference for the in-depth development of wild introduction of red deer.

Considering the ecological status of the red deer, it is being released into Huangnihe National Nature Reserve of Northeast China as the main population reinforcement to restore the red deer population and contribute to the recovery of the Amur tiger.

By analyzing and comparing the feeding composition and the gut microbial structure and function between the wild populations and the introduced populations, we explored (1) the feeding patterns of red deer under different release periods; (2) the diet-induced microbial distribution of introduced red deer; (3) the mechanism of adaption of the introduced red deer to the environmental changes based on gut microbes.

## Materials and methods

### Study area and fecal sample collection

Huangnihe National Nature Reserve is located in the south of Zhangguangcai Mountain in northeast China, with an area of 583.42 km^2^. In the early twentieth century, the red deer was widely distributed in the Huangnihe but subsequently declined, the habitat of red deer was lost and fragmented due to logging and poaching, and the red deer population was estimated to be < 10 inds (which is 0.013 density across 580 km^2^). With protection efforts since the 1990s, the population of red deer recovered but densities remain unviable and too low to support tiger recovery (< 25 inds, which is 0.043 density across 580 km^2^). With at least two Amur tigers living in this reserve, the recovery of red deer is urgent.

To promote the red deer recovery, from 2019 to 2021, we released 11 captive red deers with GPS collars in Huangnihe. The individuals released in 2019 were defined as the R2 group (*n* = 5), and those released in 2021 were defined as the R0 group (*n* = 6). Before wild release, the red deer of the R2 and R0 groups were in captivity and fed on a mixture of corn residue, soybean meal, wheat bran, corn stover, and hay, more detailed information related to the released individuals is shown in Supplementary Table 1. In addition, samples were collected from the remnant wild red deer population (*n* = 5 sampled individuals; see below), which were defined as the Wild group.

**FIGURE 1:**
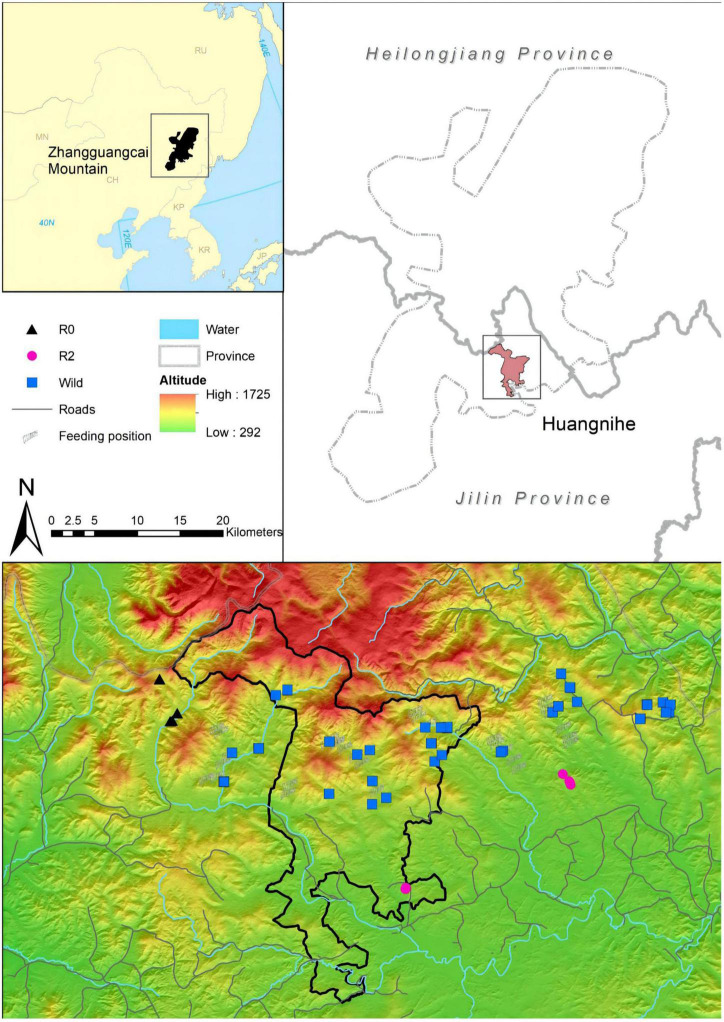
A map showing the location of the individuals of the three groups; colors represent different groups.

Fresh fecal samples were collected in December 2021. R2 and R0 groups were tracked based on the real-time data of GPS collar, and samples of Wild ones were collected outside the area of R2 and R0 (Figure 1). We collected only fresh feces attached to the surface of the snow, picked up the fecal pellets using disposable sterile gloves, and immediately stored them in a sterile cryopreservation tube, labeled them and frozen them in liquid nitrogen, and located them using handheld GPS. After sampling, it was transported to the laboratory and placed in an ultra-low temperature freezer at –80°C. A total of 16 red deer (R2 Group, *n* = 5; R0 Group, *n* = 6; Wild Group, *n* = 5) were identified from 62 samples using 10 microsatellites (Tian, 2021). The sampling covered all the individuals of R2 and R0, and more than 10% of the wild red deer population in Huangnihe.

### Deoxyribonucleic acid extraction and amplification

Plant deoxyribonucleic acid (DNA) and bacterial microbial DNA were extracted using the OMEGA Soil DNA (D5625-02) (Omega Bio-Tek, Norcross, GA, USA) kit, and the extracted plant DNA was judged by molecular size determination by 0.8% agarose gel electrophoresis (DYY-6C, Beijing), and DNA was quantified using Nanodrop (NC2000 USA).

The Plant rbcL Gene selected chloroplast rbcL gene regions of about 260 bp in length for sequencing. PCR amplification was performed using chloroplast rbcL gene-specific primers, Z1aF (5′-ATGTCACCACCAACAGAGAACTAAAGC-3′) and hp2R (5′-CGTCCTTTGTAACGATCAAG-3′). A unique barcode sequence, which is an oligonucleotide sequence of 7–10 bases, is added to the pre-primer to distinguish between different samples in the same library. The PCR uses NEB Q5 DNA high-fidelity polymerase, and the amplification system and procedure are shown in Supplementary Table 2.

The bacterial project sequenced the highly variable V3–V4 regions of the 16S rRNA gene at a length of about 468 bp. The PCR amplification selected bacterial 16S rRNA V3–V4 region-specific primers, 338F (5′-ACTCCTACGGGAGGCAGCA-3′) and 806R (5′-GGACTACHVGGGTWTCTAAT-3′). A unique barcode sequence, which is an oligonucleotide sequence of 7–10 bases, is added to the pre-primer to distinguish between different samples in the same library. The PCR uses NEB Q5 DNA high-fidelity polymerase, the system is shown in Supplementary Table 3.

### Library generation and sequencing

The library was built with the TruSeq Nano DNA LT Library Prep kit. First, end Repair Mix2 was used to excise the protruding base at the 5′ end of DNA, completed the missing base at the 3′ end, added a phosphate group at the 5′ end, then added a separate A base at the 3′ end of the DNA, added a linker with specific labels, then amplified the DNA fragments of the upper linker and purified the PCR system using BECKMAN AMPure XP beads, and finally made the final fragment selection and purification of the library by 2% agarose gel electrophoresis.

NovaSeq 6000 SP Reagent kit (500 cycles) was utilized on an Illumina NovaSeq machine for 2 × 250 bp double-ended sequencing. First, diluted the library (Index non-repeatable) gradient to 2nM, and then mixed the samples in proportion to the required amount of data. The mixed library was denatured into a single chain by 0.1N NaOH for sequencing.

### Plant data processing

The original sequence data of the plant part was decoded using the demux plugin, the cutadapt plugin was used to excise the primer, and then the sequence was spliced using the Vsearch’s fastq_mergepairs module. The fastq_filter module was used to quality control the stitching sequence. The derep_fulllength module was used to remove duplicate sequences. The deduplication sequence was clustered using the cluster_size module at the 98% similarity level and the chimera was removed using the uchime_denovo module. The perl script^1^ was then used to filter the chimeras in the sequence set after quality control to obtain high-quality sequences. The nt (2020.02 download)^2^ database in NCBI was used to annotate to compare the representative sequence with the reference sequence in the database to obtain the taxonomic information.

### Bacterial data processing

The original sequence data of the microbial part was decoded using the demux plug-in, and the qiime cutadapt trim-paired was called to excise the primer fragment of the sequence and discarded the sequence of the unmatched primer. Then, DADA2 was called through qiime dada2 denoise-paired for quality control, denoising, stitching, and de-chimerization to obtain high-quality sequences. The ASV clustering was performed by using 100% sequence consistency as the clustering criterion, and the ASV feature sequence was compared with the reference sequence in the database by the Greengenes database (Release 13.8)^3^ to obtain the taxonomic information.

### Statistical analysis

The PcoA analysis was used to describe the discreteness of feeding between the Wild, R2, and R0 groups. The difference in feeding preference between the Wild, R2, and R0 groups was compared using *t*-tests; the variance homogeneity was tested using Levine’s test, and if the test results contradicted the null hypothesis that the variance satisfies homogeneity, the Kruskal-Wallis rank test was chosen for inter-group comparison. A rarefaction curve based on the Shannon diversity index was performed to evaluate whether the sequencing depth met the requirements of the study and analysis. The PCoA analysis was performed to describe the degree of dispersion of fecal microbial structures in the Wild, R2, and R0 groups. The ANOSIM analysis based on Bray-Curtis distance metrics was used to further show the differences between groups and used random forest analysis to sort the importance of microbes with differences in abundance between groups, and screened the most critical phylum that leads to microbial structural differences between groups. The effect of feeding on the microbial structure of red deer feces was described by the PCoA-envfit analysis (different plant feeding ratios were added as environmental variables to the unconstrained coordinate axis), and the quantitative relationship between the proportion of plants fed by red deer and the microbial abundance of fecal feces was quantitatively analyzed using the Spearman Rank correlation analysis. Function clustering was carried out by PICRUSt2 function prediction to determine the main metabolic pathways of fecal microbes. The PCoA analysis was performed to detect differences in microbial function between groups and to examine metabolic pathways with differences between groups by *t*-tests. The statistical analysis was done on the R platform, and the significance criterion was *p* < 0.05, and the very significant criterion was *p* < 0.01.

## Results

### Food composition

**FIGURE 2:**
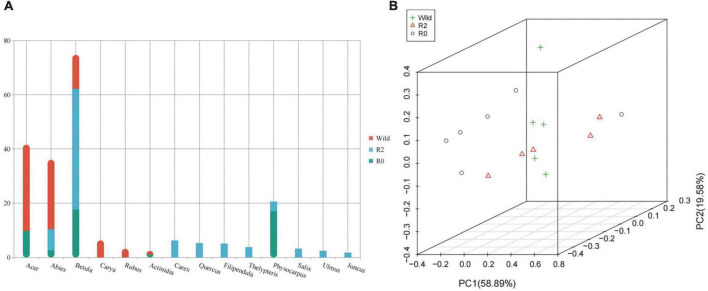
The feeding composition and structure of Wild, R2, and R0 groups. **(A)** A stacked bar graph based on the abundance of different plant genera. **(B)** The 3D PCoA plot of feeding composition based on Bray-Curtis distance metrics.

There were large differences in the composition and proportion of the main foods in the three groups (Figure 2A), of which the main food components of the Wild group included *Acer* (31.8%), *Carya* (6.3%), *Rubus* (3.2%), and *Actinidia* (1.3%). The main food components of the R2 group included *Carex* (6.3%), *Quercus* (5.3%), *Filipendula* (5.1%), *Thelypteri*s (3.8%), *Physocarpus* (3.6%), *Salix* (3.3%), *Ulmus* (2.5%), and *Juncus* (1.8%). The main food components of the R0 group included *Physocarpus* (17.0%), *Acer* (9.7%), and *Actinidia* (1.1%). The same foods among three groups included *Abies* (Wild = 25.6%, R2 = 7.8%, R0 = 2.6%) and *Betula* (Wild = 12.5%, R2 = 44.6%, R0 = 17.6%).

**FIGURE 3:**
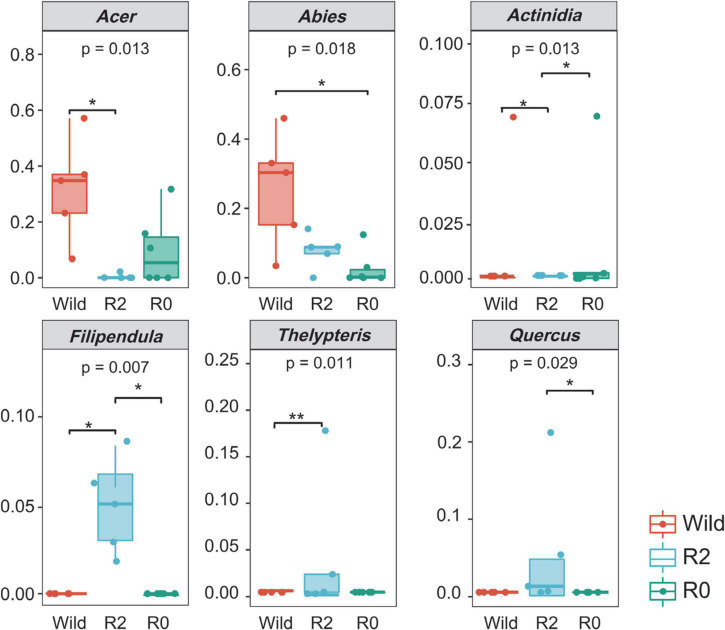
Difference in the proportion of plants consumed between three groups (*p* < 0.05) Colors represent different groups. * represents significant and ** represents very significant.

PCoA analysis showed that the samples in the group were relatively concentrated and the samples between the groups were relatively discrete, indicating that the individuals in the group ate more similarly than the individuals between the groups (Figure 2B). Comparing the feeding composition between groups, the differences between *Acer*, *Thelypteris*, *Actinidia* and *Filipendula* were found significant between the Wild group and the R2 group (*p* < 0.05), and *Quercus*, *Actinidia* and *Filipendula* were significantly different between the R2 group and the R0 group (*p* < 0.05), and *Abies* was significantly different between the Wild group and the R0 group (*p* < 0.05, Figure 3).

### Sequencing analysis and clustering

Based on 16S rRNA high-throughput sequencing technology, 1,195,310 high-quality sequences were obtained, with an average sequencing amount of 74706.9 and an average sequence length of 411.6. To determine whether the sequencing depth was sufficient for subsequent analysis, a rarefaction analysis was performed based on the Shannon diversity index, and the results showed that with the increase of the sequencing amount, the curve gradually flattened out and no longer rose, indicating that the sequencing depth met the requirements of subsequent analysis (Supplementary Figure 1).

A total of 29,649 ASVs were obtained by clustering ASVs with 100% consistency, including 7,222 ASVs in the Wild group, 7,843 ASVs in the R2 group, 13,387 ASVs in the R0 group, and 1,197 ASVs between groups. Annotation based on ASV, 29,649 ASVs belonged to 22 phyla, 48 classes, 87 orders, 157 families, and 287 genera (Supplementary Figure 2).

### Comparison of overall microbial composition and differences

The gut microbial communities of the red deer in the Huangnihe were mainly composed of Phylum Firmicutes (77.47%), Bacteroidetes (14.16%), followed by Proteobacteria (5.75%; Supplementary Figure 3). At the genus level, the > 1% abundance of the genus were *Pseudomonadaceae_Pseudomonas* (5.23%), *Roseburia* (3.24%), *Oscillospira* (3.17%), *5-7N15* (2.97%), *Ruminococcaceae_Ruminococcus* (2.57%), *CF231* (2.36%), and *Dorea* (1.57%; Supplementary Figure 4).

**FIGURE 4:**
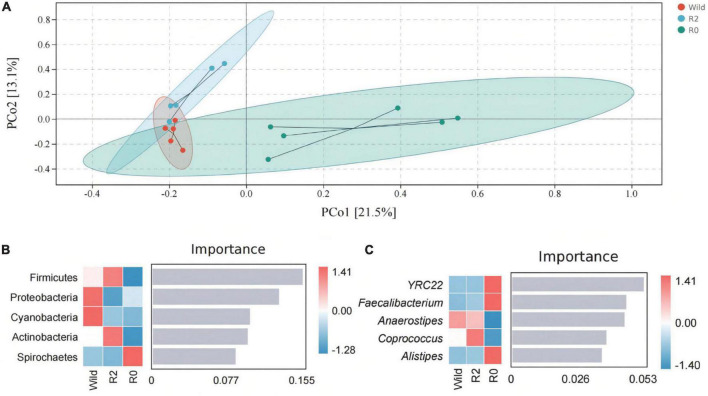
The gut microbial structure and composition of Wild, R2, and R0 group. **(A)** A PCoA plot based on Bray-Curtis distance metrics in relation to the whole dataset to describe the microbial structure between different groups. **(B)** The random forest analysis indicated the target microbial phylum with differences between groups. **(C)** Target microbial genus with differences.

The results of the PCoA analysis showed that the fecal microbial structure in the R2 group has some similarities with that in the wild group, while the microbial communities in the R0 were quite different (Figure 4A). The ANOSIM analysis further showed the significant microbial differences between groups (Wild vs. R0: *R* = 0.419, *p* = 0.013; R2 vs. R0: *R* = 0.451, *p* = 0.005; Supplementary Table 4). Through random forest analysis, it was found that the microbes of Firmicute and Proteobacteria had significant differences in abundance between the three groups (*Importance* > 0.1), which were the main phyla causing differences in microbial communities between groups (Figure 4B). *YRC22*, *Faecalibacterium*, and *Anaerostipes* were the main genera responsible for differences in microbial communities between groups (*Importance* > 0.04; Figure 4C).

### Relationship between feeding and the gut microbial communities

**FIGURE 5:**
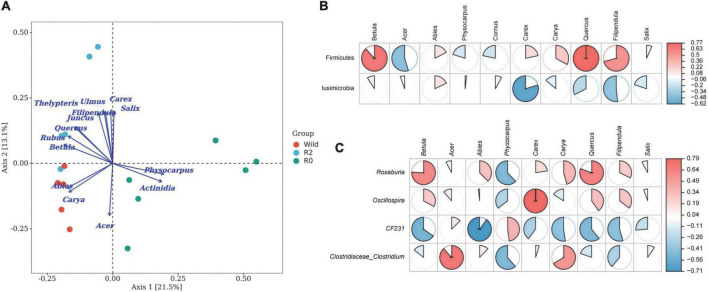
The relationship between feeding plants and gut microbes. **(A)** The PCoA plot based on environment variables fitted to the unconstrained coordinate axis; dots represent the microbial community, arrows represent plant species, dot color represents different groups. **(B)** Microbial phylum associated with feeding. **(C)** Microbial genus associated with feeding.

The results of the PCoA-envfit analysis showed that the species of feeding plants had a significant influence on the microbial structure; *Abies*, *Carya*, and *Acer* had a significant influence on the microbial community structure of the Wild group, and *Betula*, *Quercus*, *Salix*, *Ulmus*, *Filipendula*, *Juncus*, *Carex*, and *Thelypteris* had a significant influence on the microbial community structure of the R2 group, and *Actinidia* and *Physocarpus* had a significant influence on the structure of the R0 microbial communities (Figure 5A).

The results of the Spearman Rank correlation analysis showed that the feeding was mainly related to the abundance of Firmicute, and the feeding intensity of *Betula* and *Quercus* had a very significant positive correlation with Firmicute (*p* < 0.01), and the feeding intensity of *Filipendula* was significantly positively correlated with the Phylum Firmicute (*p* < 0.05; Figure 5B). At the genus level, *Roseburia*, *Oscillospira*, and *Clostridiaceae_Clostridium* were positively correlated with the main food components of *Betula*, *Acer*, *Quercus*, etc. (*p* < 0.05), which might be potentially important microbial communities for handling complex food resources (Figure 5C).

### Comparison of microbial functions between groups

**FIGURE 6:**
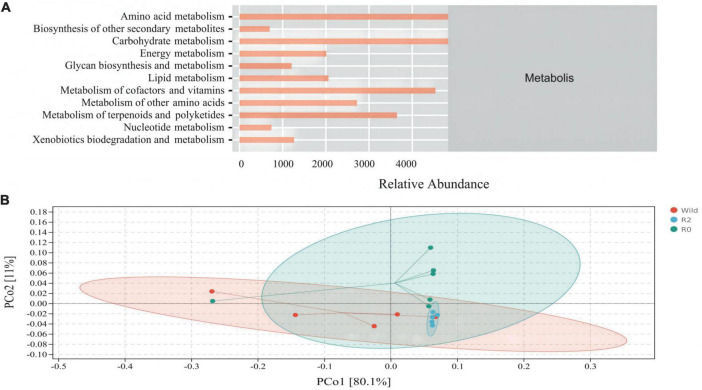
The composition and structure of main microbial functions. **(A)** An abundance distribution plot of secondary function pathways in Metabolis according to PICRUSt2 function prediction. **(B)** A PCoA plot based on Bray-Curtis distance metrics associated with function dataset; colors represent sample sets of microbial functions (PCo1 = 80.1%, PCo2 = 11%).

PICRUSt2 function prediction was performed on gut microbial communities, metabolism into the most important pathways for microbial clustering (77%), including amino acid metabolism (17.3%), carbohydrate metabolism (17.0%), metabolism of cofactors and vitamins (15.8%), and metabolism of terpenoids and polyketides (12.7%) made up its most dominant secondary pathway (Figure 6A).

**FIGURE 7:**
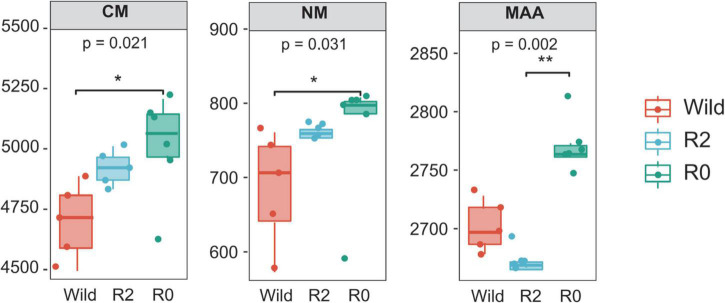
The microbial functions and groups with significant differences (*p* < 0.05), CM, Carbohydrate metabolism; NM, Nucleotide metabolism; MAA, Metabolism of other amino acids. * represents significant and ** represents very significant.

PCoA analysis found that microbial function clusters were more similar between the R2 and R0 groups, while microbial function clusters were more different from those in the Wild group (Figure 6B). To further explore the differences in microbial function between groups, the difference analysis of metabolic pathways between groups was performed, and it was found that the carbohydrate metabolism pathway and nucleotide metabolism pathway were significantly different between the Wild group and the R0 group (*p* < 0.05), and the R0 group was significantly higher than the Wild group. The metabolism of other amino acids pathway differed significantly between the R2 group and the R0 group (*p* < 0.05), and the R0 group was significantly higher than the R2 group (Figure 7).

## Discussion

This study compared the feeding habitats and gut microbes of wild and reinforcement red deer in the Huangnihe National Nature Reserve. The feeding patterns of red deer between the wild and reinforced populations and the synergistic relationship with the gut microbes were analyzed, and it was proved that there were different feeding strategies between the wild population and the introduced populations. The microbial communities made corresponding changes to adapt to feeding. To a certain extent, the wild release reshaped their gut microbial structure and made it integrate into the direction of the wild population. The period since release into the wild is the key factor in reshaping the structure of the intestinal flora. The microbial communities play a key role in the adaptation of introduced red deer to the natural environment, especially in ensuring the nutritional supply of introduced red deer and improving the adaptability to complex environmental conditions.

Supplementary feeding was set up within Huangnihe to help them endure the winter with less food. However, the feeding preferences between R2 and R0 were different, and no artificially supplied food ingredients, such as corn flour (*Zea mays*) and hay, were found in the food composition of the three groups. While sampling in the field, we did not find red deer footprints around the feeding site, indicating that introduced individuals did not have a great dependence on supplemental foods. Considering that the main components of food components of introduced red deer were natural foods, and the foods may not be a limiting factor in winter due to a small number of red deer, we infer that introduced red deer should have a certain adaptability and processing capacity to natural food resources. It could be seen from the feeding patterns that the Wild group has formed a specific feeding strategy adapting to the natural environmental conditions, showing a preference for *Acer* and *Abies*, while the R2 group, which has been released for 2 years, has a strong preference for *Betula*. When sampling, we found the vegetation type in the R2 group habitat was mainly *Betula*-*Quercus* Forest and *Betula*-*Abies* forest. We speculated that the preference for *Betula* was related to its availability and palatability. R0 has just been released to the wild, their diets were more mixed, and the types of feeding plants were as high as dozens of genera, a nutritional strategy with a pronounced feeding preference for one or more plants similar to the Wild and R2 groups have not yet been formed.

Food can directly affect the intestinal microbial community, while the intestinal microbiome plays an important role in host nutrition (Ley et al., 2008; Wu et al., 2017; Li G. et al., 2019; Huang et al., 2021). We demonstrate the role of diet in shaping the intestinal microbial community in wild and released red deer in northeast China. According to the results of PCoA-envfit analysis, it could be seen that plant species directly performed impacts on the microbial structure, and the proportion of feeding mainly affected the abundance of Firmicutes. For introduced red deer, the gut microbiome adapted to changes in food resources and the environment by adjusting the microbial structure and increasing the abundance of some microbial communities related to food digestion, such as phylum Firmicutes, genera *Roseburia*, *Oscillospira*, and *Clostridiaceae_Clostridium*. The phylum Firmicutes carries a large number of enzymes encoding genes for energy metabolism and can produce a variety of digestive enzymes to help the host degrade various substances in food, such as structural and non-structural carbohydrates (Middelbos et al., 2010; Zhao et al., 2018). In addition, the genera *Roseburia*, *Oscillospira*, and *Clostridiaceae_Clostridium* belonged to the Firmicutes, and are important bacteria that ferment complex carbohydrates and play an important role in the metabolism of nutrients, such as carbohydrates and fats (Konikoff and Gophna, 2016; Tamanai-Shacoori et al., 2017; Diether and Willing, 2019). It is worth noting that although feeding led to some differences in microbial community between groups, the microbial structure in R2 was closer to the wild, which indicated that there were other factors affecting the microbial structure in addition to food. Environmental factors are known to play a more important role in shaping the structure of the gut microbiota (Jin et al., 2021; Gacesa et al., 2022). In this study, R2 experienced a long field adaptation period, and R0 had just been released into the wild from the captive environment. The R2 group and the Wild group lived in similar environments for a long time, resulting in the transformation of the microbial structure of the R2 group to more similar to the Wild group. This was significantly different from the microbial structure of the R0 group.

It was worth noting that the functional pathway composition of the R2 group and the R0 group in the study were more similar, which were very different from the Wild group, and the results of microbial functional clustering were exactly opposite to the microbial structure. On the one hand, changes in the microbial composition and structure did not necessarily lead to changes in their functions, and different microbial communities might perform similar or same functions (Phillips et al., 2017). On the other hand, the microbial functions of introduced populations might be more stable. The study of Tibetan wild ass (*Equus kiang*) showed that the captive ass exhibited relatively stable microbial function in the face of changes in food and the environment (Gao et al., 2020). We speculated that because both R2 and R0 of red deer were captive-bred animals before release, their microbial function might not change easily, and changes in food and environment did not have a significant impact on their microbial function. By comparing the differences in functional abundance between groups, it was found that the abundance of carbohydrate metabolism, nucleotide metabolism, and metabolism of other amino acid pathways of the R0 group was significantly higher than those of the other two groups. Most of the food components of plant-eating animals are plant cell wall polysaccharides, the host itself does not have the enzyme to digest polysaccharides, while the gut microbiome can degrade the resistant starch and non-starch polysaccharides in plants, using them as carbon sources and energy sources for themselves and the host (Gill et al., 2006; Flint et al., 2012). The carbohydrate metabolism pathway formed an important metabolic pathway for the R0 group, helping to process complex food resources in the natural environment. Dietary proteins and their metabolite amino acids are essential nutrients for organisms, gut microbes can mediate protein metabolism and host immune responses, and are widely involved in the metabolism and utilization of nitrogen nutrients (Oliphant and Allen-Vercoe, 2019; Zhao et al., 2019), and elevated metabolism of other amino acids pathways might be associated with crude proteins in plants. Purine nucleotides are important energy substances in the body, of which ATP is a direct energy source for bone contraction (Witte and Herde, 2020), and nucleotide metabolism might be associated with the treatment of complex environmental conditions and the maintenance of body temperature in winter.

## Conclusion

This study provides an understanding of the adaption process of captive red deer translocated into the wild as part of a conservation reinforcement with regard to the role of the gut microbial community in coping with foods and the environment. In summary, introduced individuals of red deer in Huangnihe have a certain ability to process natural foods and adapt to novel field environments, and the wild environmental conditions have reshaped the intestinal microbial structure of introduced individuals so that the microbial structure is integrated into the wild population. The phylum Firmicutes and multiple genera could help introduced red deer cope with complex food resources in the wild, ensuring their nutritional supply of them and getting rid of the influence of artificial supplementary feeding, improving their health, and greatly increasing the effectiveness of adaption.

## Data availability statement

The datasets presented in this study can be found in online repositories. The data of plant rbcL gene presented in the study are deposited in: https://www.ncbi.nlm.nih.gov/, accession number PRJNA861951. The data of 16S rRNA gene presented in the study are deposited in: https://www.ncbi.nlm.nih.gov/, accession number PRJNA861708.

## Ethics statement

Ethical review and approval was not required for the animal study because we just selected fecal samples of red deer as research materials, it does not cause any harm to animals.

## Author contributions

XT collected fecal samples and extracted DNA. JG carried out the statistical analysis work and article writing work. HB participated in data analysis and article revision. YSu and TG participated in the revision of the article. YSo collected field and experimental data. YJ and MZ participated in the revision of the article and provided research funds. All authors read and approved the final version of the manuscript.

## Footnotes

1
https://github.com/torognes/vsearch/wiki/VSEARCH-pipeline

2
https://www.ncbi.nlm.nih.gov/home/download/

3
http://greengenes.secondgenome.com/

## Supplementary material

Supplementary Figure 1 The rarefaction curve based on the Shannon diversity index; the horizontal axis represents the sequencing depth, and different colors represent samples.

Supplementary Figure 2 A Venn plot based on the number of ASVs between Wild, R2, and R0 groups.

Supplementary Figure 3 A pie chart of the gut microbial composition of red deer on phylum level.

Supplementary Figure 4 A pie chart of the gut microbial composition of red deer on genus level.
