# Supplementary material for: Diet-induced microbial adaptation process of red deer (Cervus elaphus) under different introduced periods
Source: Front Microbiol. 2022 Oct 20;13:1033050. doi: 10.3389/fmicb.2022.1033050 (PMC9632493; doi:10.3389/fmicb.2022.1033050)
Supplement: Supplementary file 1 [file Table_1.DOCX]

**Table. S1 The information of released individuals of red deer**

| **Num** | **Groups** | **Gender** | **Age** | **Main**  **foods** | **Daily**  **intake** | **Feeding frequency** |
| --- | --- | --- | --- | --- | --- | --- |
| 1 | R2 | ♀ | 3 | Corn, soybean meal, wheat bran, hay | 1 catty/ind | Twice a day |
| 2 | R2 | ♀ | 3 | Corn, soybean meal, wheat bran, hay | 1 catty/ind | Twice a day |
| 3 | R2 | ♀ | 3.5 | Corn, soybean meal, wheat bran, hay | 1 catty/ind | Twice a day |
| 4 | R2 | ♀ | 2.5 | Corn, soybean meal, wheat bran, hay | 1 catty/ind | Twice a day |
| 5 | R2 | ♂ | 3 | Corn, soybean meal, wheat bran, hay | 1-1.5 catty/ind | Twice a day |
| 6 | R0 | ♀ | 3.5 | Corn, soybean meal, wheat bran, hay | 1 catty/ind | Twice a day |
| 7 | R0 | ♀ | 3 | Corn, soybean meal, wheat bran, hay | 1 catty/ind | Twice a day |
| 8 | R0 | ♀ | 3 | Corn, soybean meal, wheat bran, hay | 1 catty/ind | Twice a day |
| 9 | R0 | ♀ | 3 | Corn, soybean meal, wheat bran, hay | 1 catty/ind | Twice a day |
| 10 | R0 | ♀ | 3 | Corn, soybean meal, wheat bran, hay | 1 catty/ind | Twice a day |
| 11 | R0 | ♂ | 3 | Corn, soybean meal, wheat bran, hay | 1-1.5 catty/ind | Twice a day |
