# Supplementary material for: Diet-induced microbial adaptation process of red deer (Cervus elaphus) under different introduced periods
Source: Front Microbiol. 2022 Oct 20;13:1033050. doi: 10.3389/fmicb.2022.1033050 (PMC9632493; doi:10.3389/fmicb.2022.1033050)
Supplement: Supplementary file 2 [file Table_2.DOCX]

**Table. S2 The amplification system of PCR based on plant gene**

| **Composition** | **Volum (μL)** |
| --- | --- |
| Q5 high-fidelity DNA polymerase | 0.25 |
| 5*Reaction Buffer | 5 |
| 5* High GC Buffer | 5 |
| dNTP（10mM） | 2 |
| Template DNA | 2 |
| Forward primers（10uM） | 1 |
| Reverse primers（10uM） | 1 |
| water | 8.75 |

**Procedure：**

98℃ 5min

98℃ 30 s

52℃ 30 s 27 cycles

72℃ 45 s

72℃ 5 min

12℃ ∞
