# Supplementary material for: Diet-induced microbial adaptation process of red deer (Cervus elaphus) under different introduced periods
Source: Front Microbiol. 2022 Oct 20;13:1033050. doi: 10.3389/fmicb.2022.1033050 (PMC9632493; doi:10.3389/fmicb.2022.1033050)
Supplement: Supplementary file 4 [file Table_4.DOCX]

**Tab. S4 The results of Anosim analysis based on bray cuitis**

| **Group1** | **Group2** | **Sample size** | **Permutations** | ***R*** | ***p*-value** |
| --- | --- | --- | --- | --- | --- |
| Wild | R0 | 11 | 999 | 0.418667 | 0.013 |
| R2 | R0 | 11 | 999 | 0.450667 | 0.005 |
