# Supplementary figures and images for: Diet-induced microbial adaptation process of red deer (Cervus elaphus) under different introduced periods
Source: Front Microbiol. 2022 Oct 20;13:1033050. doi: 10.3389/fmicb.2022.1033050 (PMC9632493; doi:10.3389/fmicb.2022.1033050)

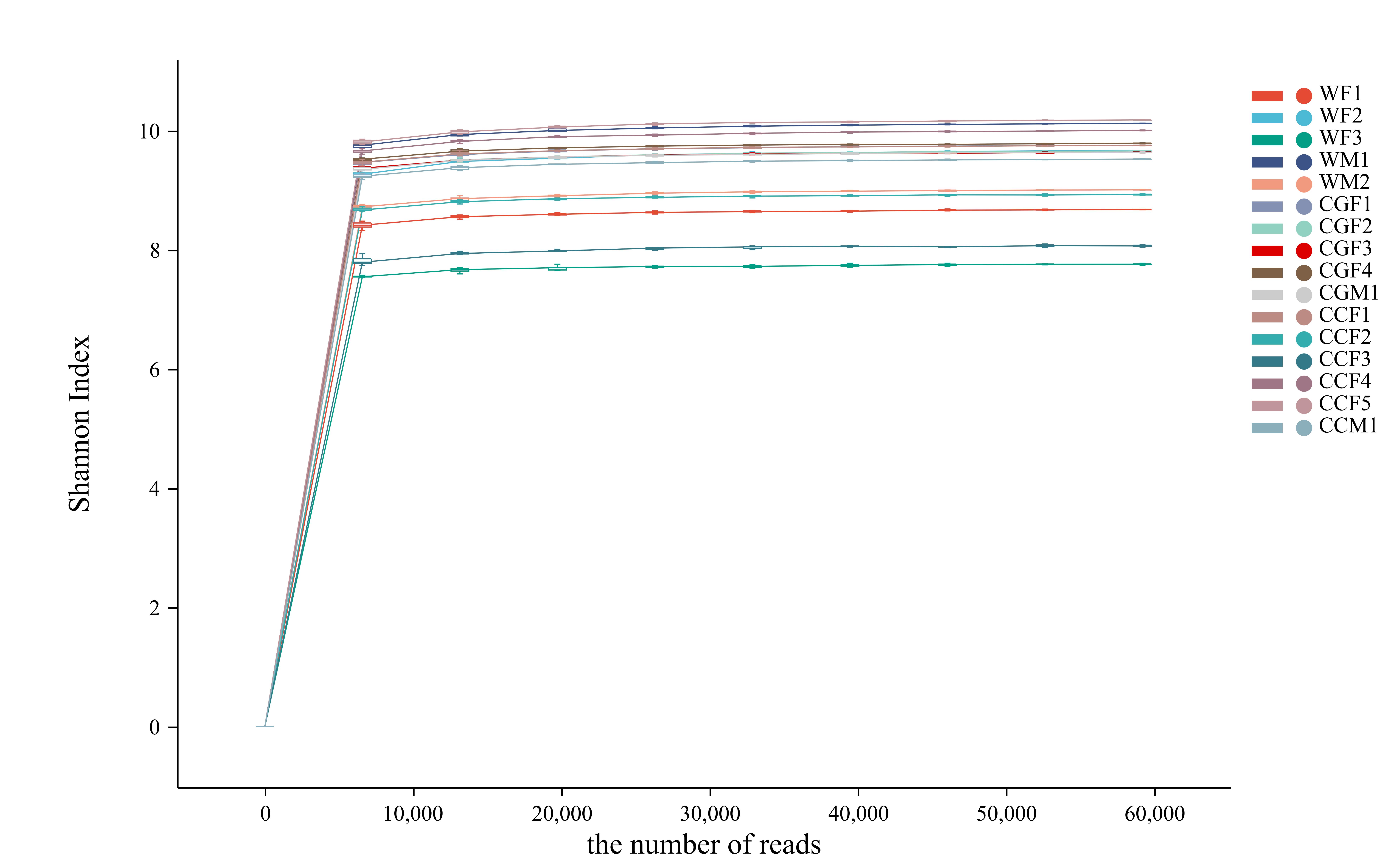

Supplement: Supplementary Figure 1 — The rarefaction curve based on the Shannon diversity index; the horizontal axis represents the sequencing depth, and different colors represent samples. [file Image_1.JPEG]

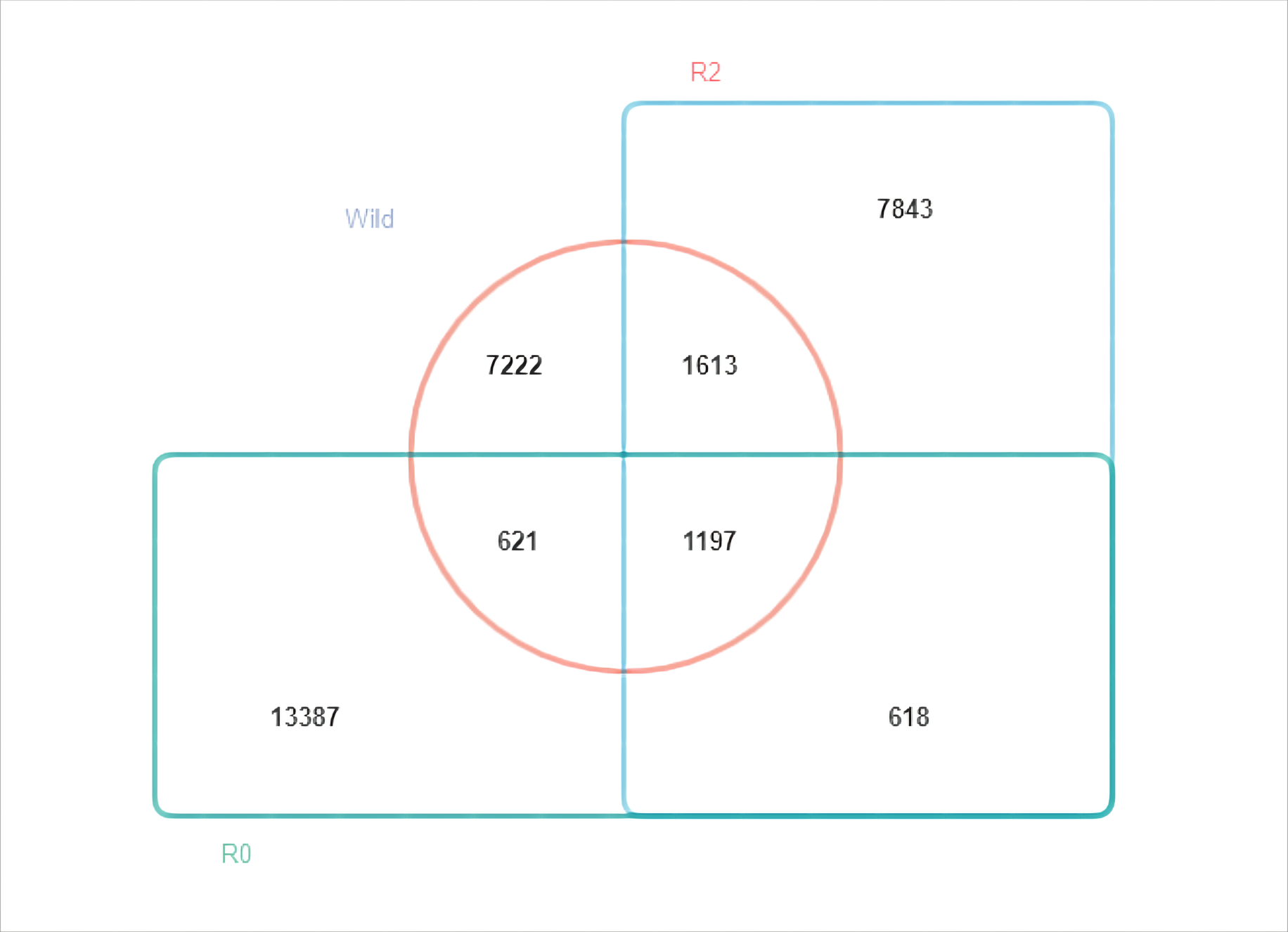

Supplement: Supplementary Figure 2 — A Venn plot based on the number of ASVs between Wild, R2, and R0 groups. [file Image_2.PNG]

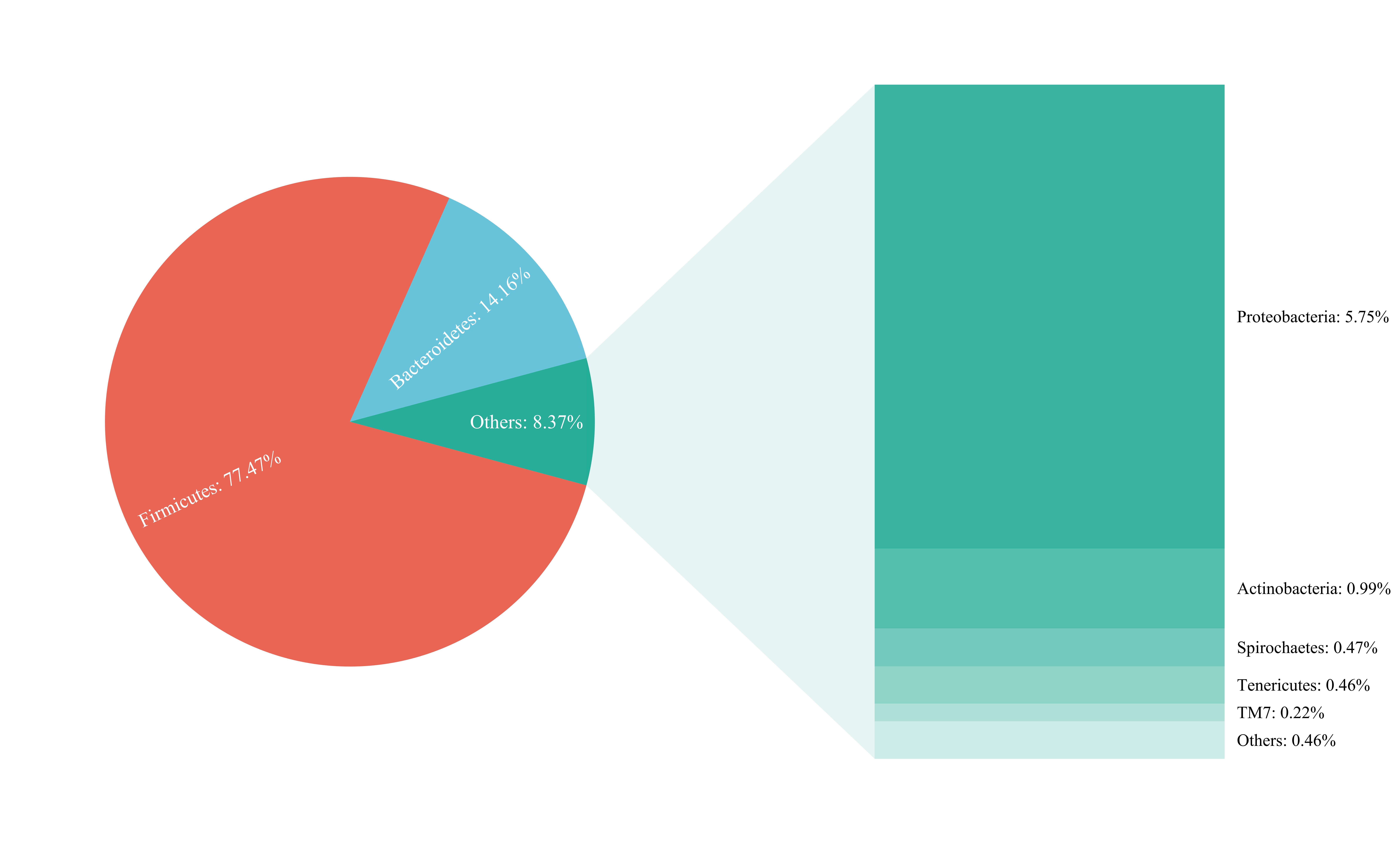

Supplement: Supplementary Figure 3 — A pie chart of the gut microbial composition of red deer on phylum level. [file Image_3.JPEG]

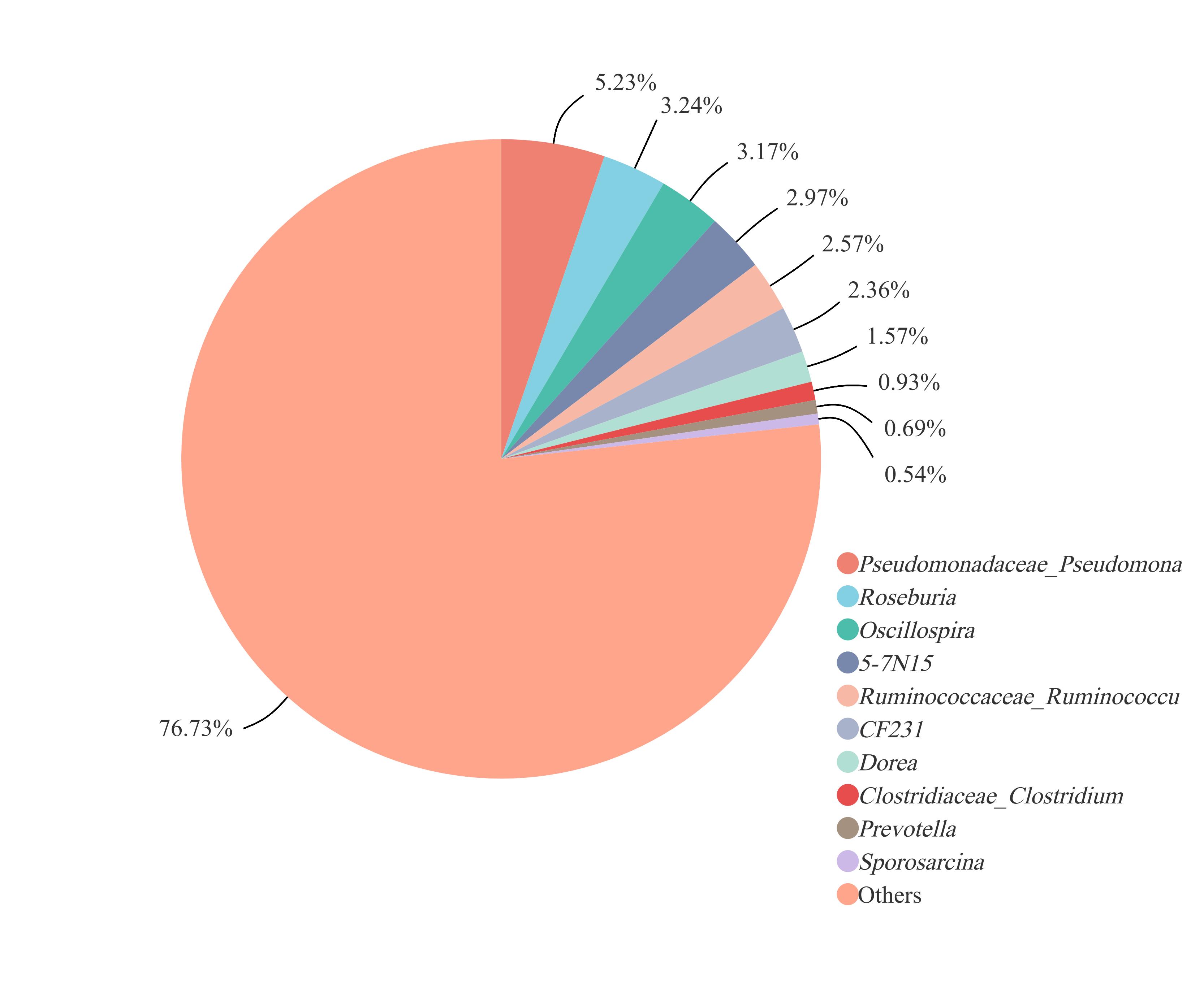

Supplement: Supplementary Figure 4 — A pie chart of the gut microbial composition of red deer on genus level. [file Image_4.jpg]
